# A novel nanomicelle based on Rebaudioside A: An oral nanoplatform with enhanced nephroprotective effect of myricetin

**DOI:** 10.1016/j.ijpx.2025.100389

**Published:** 2025-09-08

**Authors:** Tian Wang, Zhen Gao, Chuanlong Guo, Wenyong Zhu

**Affiliations:** a Department of Thoracic Surgery, Qilu Hospital (Qingdao), Cheeloo College of Medicine, Shandong University, No. 758 Hefei Road, Qingdao 266035, China; b Department of Thoracic Surgery, Jiaozhou Central Hospital of Qingdao, No.99 South Yunxihe Road, Qingdao 266300, China; c Department of Pharmacy, College of Chemical Engineering, Qingdao University of Science and Technology, No. 53 Zhengzhou Road, Qingdao 266042, China

**Keywords:** Cisplatin, Myricetin, Acute kidney injury, DNA damage, cGAS-STING

## Abstract

Cisplatin-induced acute kidney injury (AKI) is a significant clinical challenge, primarily characterized by inflammatory responses and oxidative stress. This study aimed to develop a myricetin (Myr) loaded Rebaudioside A (RA) nanomicelle delivery system (RA-Myr) and investigate its nephroprotective effects both *in vitro* and *in vivo*. RA-Myr nanomicelles were prepared using a thin film hydration method. The characterization of RA-Myr included evaluating particle size, encapsulation efficiency, and stability. The antioxidant capacity of RA-Myr was assessed using the FRAP assay, and cellular uptake was evaluated using coumarin 6-loaded RA nanomicelles. The protective effects and potential mechanisms of RA-Myr on cisplatin-induced AKI were studied in HK-2 cells and male Kunming mice. RA-Myr significantly inhibited cisplatin-induced suppression of HK-2 cell proliferation, reduced ROS accumulation, and restored mitochondrial membrane potential. *In vivo*, RA-Myr alleviated cisplatin-induced AKI, evidenced by decreased blood urea nitrogen (BUN) and serum creatinine (SCr) levels, and mitigated kidney tissue pathological damage. Mechanistically, RA-Myr protected against cisplatin-induced DNA damage and inhibited the cGAS-STING pathway. The RA-Myr nanomicelle delivery system shows promise as a potential strategy for alleviating cisplatin-induced AKI by enhancing the nephroprotective effects of Myr.

## Graphical abstract

**Figure:**
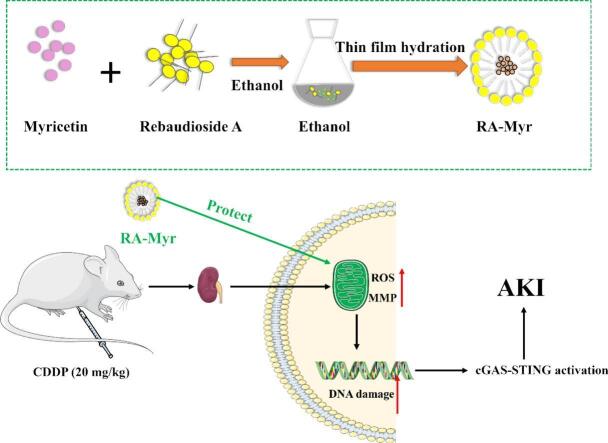

## 1 Introduction

Acute kidney injury (AKI) is a common clinical disease characterized by a decline in kidney function (Peerapornratana et al., 2019). Multiple factors may lead to the occurrence of AKI, such as invasive surgery and the use of nephrotoxic drugs like cisplatin, gentamicin, and contrast agents (Holditch et al., 2019). Cisplatin is an important platinum-containing chemotherapy drug widely used to treat solid tumors, including bladder cancer, non-small cell lung cancer, breast cancer, and malignant melanoma (Su et al., 2022). However, studies have found that 20 % to 35 % of cisplatin chemotherapy patients have a risk of developing nephrotoxicity and leading to AKI (Dupre et al., 2016). The pathogenesis of cisplatin induced AKI is currently unclear, and its possible mechanisms include inducing inflammatory reactions and oxidative damage (Casares et al., 2012; Dugbartey et al., 2016). Unfortunately, there are currently no effective drugs for the prevention and treatment of AKI in clinical practice.

The cyclic guanylate adenylate synthase (cGAS) - stimulator of interferon gene (STING) pathway is a key signal that regulates inflammatory responses in infection, cellular stress, and tissue damage. cGAS, as a DNA receptor, can recognize both intracellular and extracellular sources of DNA such as pathogen DNA, as well as cellular DNA such as nuclear DNA and mitochondrial DNA (mtDNA) (Decout et al., 2021; Skopelja-Gardner et al., 2022). The activated cGAS further activates STING and downstream pathways, such as the inflammatory factors, ultimately inducing the body's immune response (Zhang et al., 2020). In the context of cisplatin-induced kidney injury, cisplatin causes significant DNA damage, including the formation of DNA adducts and double-strand breaks (DSBs) (Yimit et al., 2019). This DNA damage leads to the release of damaged DNA into the cytosol, where it is recognized by cGAS, thereby activating the cGAS-STING pathway (Maekawa et al., 2019a). The activation of this pathway contributes to the inflammatory response and exacerbates kidney injury. Recent studies have found that the occurrence of kidney injury may be related to the activation of the cGAS/STING pathway (Gao et al., 2022), demonstrating cGAS-STING inhibition alleviates cisplatin-induced AKI.

Myricetin (Myr, 3,3′,4′,5,5′, 7-hexahydroxyflavones), widely present in most berries, vegetables, and various herbs, and with excellent biological activities such as anti-inflammatory, antioxidant, *etc.* (Li et al., 2022; Sun et al., 2021). Studies have confirmed the potential of Myr in the treatment of diabetes nephropathy and non-alcoholic fatty liver (Aminzadeh and Bashiri, 2020). However, its clinical translation is hindered by poor water solubility (<5 μg/mL) and low oral bioavailability (<10 %), limiting therapeutic efficacy (El-Gendy et al., 2024). Rebaudioside A (RA) is a steviol glycoside extracted from the natural plant *Stevia rebaudioides*. RA is a diterpene with both hydrophobic and hydrophilic groups, allowing it to form self-assembled micelles (SAMs) in water. Such micelles are widely used in food and pharmaceuticals to protect compounds from degradation in the gastrointestinal environments and improve their solubility and bioavailability (Liu et al., 2025; Wang et al., 2020).

In our previous studies, we prepared Kolliphor HS15 micelles loaded with Myr (HS15-Myr) and demonstrated their nephroprotective effects in preliminary *in vitro* and *in vivo* studies (Qi et al., 2023). However, several limitations were identified. Firstly, the exact mechanism, particularly in relation to the cGAS-STING pathway, remained unclear. Secondly, the long-term stability and biodistribution of the nanomicelles *in vivo* required further investigation. Additionally, the therapeutic potential in a clinically relevant model of cisplatin-induced AKI had not been fully explored. Therefore, this study aims to address these gaps by investigating the protective effects of RA-Myr on cisplatin-induced AKI, focusing on its impact on DNA damage and the cGAS-STING pathway, as well as evaluating its long-term stability and biodistribution.

## 2 Material and method

### 2.1 Materials

RA (purity >98 %) was purchased from Jining Aoxing Stevia Products Co., Ltd. (Jining, China). Myr (purity >98 %) and coumarin 6 (Cou 6, purity >98 %) were purchased from aladdin (Shanghai, China). 3-(4,5-dimethylthiazol-2-yl)-2,5-diphenyltetrazolium bromide (MTT), 2,7-Dichlorodihydrofluorescein diacetate (DCFH-DA), JC-1 were purchased from Beyotime Biotechnology (Shanghai, China). Serum Creatinine (C011-2-1) Assay kit and blood urea nitrogen (C013-2-1) Activity Kit were obtained from Nanjing Jiancheng Bioengineering Institute (Nanjing, China). Proteintech (Wuhan, China) provided Histone H2A.X (10856-1-AP), cGAS (26416-1-AP) and TMEM173/STING (19851-1-AP). And the secondary antibodies (ASS1009) were obtained from ABGENT (Suzhou, China).

Human renal proximal tubule (HK-2) cells were purchased from the BeNa Culture Collection (Henan, China). HK-2 cells were cultured in RPMI-1640 medium supplemented with high glucose and 10 % (*v*/v) FBS, incubated in a humidified 5 % CO_2_ incubator at 37 °C. The aim of this study is to investigate the protective effect of RA-Myr on cisplatin induced AKI. The cisplatin induced HK-2 cell injury model is a classic *in vitro* kidney injury model (Lu et al., 2020).

### 2.2 Preparation and of characterization the RA-Myr nanomicelles

RA-Myr nanomicelles were prepared using thin film hydration assay, and the characterization and evaluation methods were as previously reported (Gao et al., 2022; Qi et al., 2023; Wang et al., 2020). Briefly, Myr (10 mg) and RA (with different RA/Myr weight ratio) were dissolved in ethanol. The solvent slowly evaporated using a rotary evaporator under reduced pressure at 40 °C until a dry film was formed on the inner wall of the flask (This layer of dry film is removed and stored at room temperature). Phosphate buffer solution (PBS) was added, and the flask was rotated under normal pressure for about 10 min by using a rotary evaporator at 120 rpm to obtain micelle dispersion. The unencapsulated Myr was filtered through a 0.22 μm filter.

The Myr content in RA-Myr nanomicelles was measured at 375 nm using a UV–visible spectrophotometer. Briefly, RA-Myr nanomicelles were divided into two groups: one group is unfiltered (solution A), and the other group is filtered with 0.22 μM filter (solution B). Then, the absorbance values of the two solutions at 375 nm were detected.

Entrapment Efficiency was calculated accordingly to the following equation:

$$\text{Entrapment Efficacy}\;\left(\%\right)=\frac{\mathrm{Weight of}\;\mathrm{Myr}\;\mathrm{in solution}\;B}{\mathrm{Weight ofMyr in}\;\mathrm{solution}\;A}\times 100\%$$

The particle size distribution and zeta potential were measured by dynamic light scattering (DLS) instrument (ZetaPALS; Malvern Instruments Co., Ltd.). The nanostructure of the RA-Myr nanomicelles were observed by transmission electron microscopy (TEM) (Gao et al., 2022).

### 2.3 Stability of RA-Myr nanomicelles

To evaluate the stability of RA-Myr nanomicelles, the particle size of RA-Myr nanomicelles (1 mg/mL) were measured. The sample was stored in powder form (25 °C) for 8 month (60 % relative humidity) and dissolved in ultrapure water (pH 6.8) during testing. Particle size was measured monthly.

### 2.4 *In vitro* release study

The *in vitro* release properties of the RA-Myr in simulated gastric and simulated intestinal fluids were analyzed using a previously reported dialysis method (Liang et al., 2021). Briefly, 1 mL of the prepared RA-Myr (5 mg/mL) were placed in a dialysis bag (3500 Da), which was then shaken in gastric phase (pH 1.5) for 2 h and small intestine stage (pH 7.5) for another 6 h. The samples were detected by UV spectrophotometry, and the concentration was calculated. All experiments were performed in triplicate.

### 2.5 *In vitro* antioxidant assay

The antioxidant capacity of RA-Myr nanomicelles was detected by FRAP assay as previously reported (Qi et al., 2023). Briefly, RA, free Myr or RA-Myr nanomicelles (with various concentrations) solutions were mixed with freshly prepared FRAP solutions and kept at room temperature for various times (15 min, 30 min, 60 min, 90 min, and 120 min). The absorbance at 595 nm was measured using a microplate reader. All experiments were performed in triplicate.

### 2.6 Cellular uptake of RA nanomicelles loaded with coumarin 6 (Cou6)

To investigate the cellular uptake of RA delivery systems, we prepared RA nanomicelles loaded with coumarin 6 (RA-Cou6) using thin film hydration method as described in section 2.2. HK-2 cells were treated with RA-Cou6 (with Cou6 concentration of 50 μg/mL) for 3 min, 5 min, 15 min, and 30 min. Cells were photographed under a fluorescence microscope.

### 2.7 Cell viability assay

HK-2 cells were seeded in 96-well plates and incubated overnight. Cells were treated with free Myr or RA-Myr nanomicelles for 1 h, then cells were treated with cisplatin (20 μM) for 24 h. After treatment, cell viability was measured using MTT assay (Xue et al., 2024).

### 2.8 DCFH-DA and JC-1 staining assay

HK-2 cells were seeded into 48-well plates and cultured overnight. The cells were treated with RA-Myr nanomicelles (10 μM) for 1 h and then treated with cisplatin for another 24 h. ROS accumulation and mitochondrial membrane potential (MMP) were detected by DCFH-DA and JC-1 labeling, respectively.

### 2.9 Immunofluorescence assay

The effect of RA-Myr on DNA damage and cGAS/STING pathway was detected using immunofluorescence assay, as previously reported (Qi et al., 2023). HK-2 cells were inoculated into 48 well plates and cultured overnight. The cells were treated with RA-Myr nanomicelles (10 μM) for 1 h and then treated with cisplatin for another 24 h. After the treatment, cells were fixed with 4 % paraformaldehyde for 15 min and then blocked with 5 % nonfat dry milk for 1 h. The cells were incubated with primary antibody at 4 °C overnight. After washing TBST three times, the cells were incubated with fluorescent labeled secondary antibodies at room temperature for 1 h. After washing, the cells were stained with DAPI for 10 min and finally photographed under a fluorescence microscope.

### 2.10 Animal study and sample collection

**Fig. 1:**
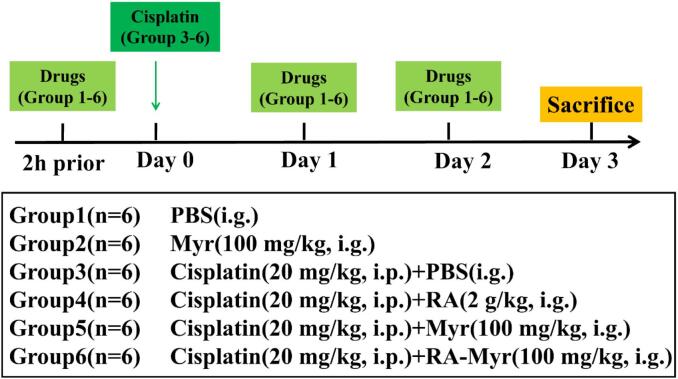
*In vivo* experimental grouping and administration protocols.

Male Kunming mice (4 weeks of age) were obtained from Daren Fucheng Experimental Animal Breeding Co., Ltd. The animal experiments in this research work complied with the ARRIVE guidelines and were conducted as per the U.K. Animals (Scientific Procedures) Act, 1986, and associated guidelines. The animal study was approved by the Qingdao University of Science and Technology Ethics Committee for Animal Experimentation (approval document no. QKDLL2024–10, Qingdao, China). The *in vivo* experimental design and animal procedures were shown in Fig. 1. cisplatin (20 mg/kg, i.p.) was selected based on established protocols for AKI induction, balancing nephrotoxicity severity with animal welfare (Holditch et al., 2019). On the first day, cisplatin was administered intraperitoneally to the mice, followed by corresponding treatment by gavage. After 3 days, the mice were anesthetized with carbon dioxide, blood (from retro-orbital, 0.5 mL) and tissues were collected immediately. One kidney was rinsed in PBS, flash-frozen in liquid nitrogen, and stored at −80 °C, and one was fixed with neutral formaldehyde for pathological study. Myricetin (100 mg/kg) was chosen as it achieves therapeutic plasma/tissue levels without toxicity in murine AKI models (Qi et al., 2023).

### 2.11 Serum biochemistry

The serum was separated by centrifuging the blood at 11,000 rpm for 15 min. The blood urea nitrogen (BUN) and serum creatinine (SCr) levels in the serum were measured using commercially available diagnostic kits according to the manufacturer's instructions (Qi et al., 2023).

### 2.12 Real-time fluorescence quantitative reverse transcriptase polymerase chain reaction (RT-qPCR)

A whole kidney was homogenized for RNA extraction using TRIzol (Thermo Fisher Scientific, Waltham, MA, USA) as described previously (Liu et al., 2023). RNA quality was confirmed *via* spectrophotometry (A260/A280 ratio > 1.8) and transformed to cDNA. Quantitative PCR analysis was then performed using SYBR green, and the changes in expression were calculated by using the 2^- ΔΔCT^ method. The primers used for PCR are shown in Table 1.

**Table 1:** primers information.

Gene	Forward primer	Reverse primer
m-SOD	CGGCCTACGTGAACAATCTCA	GGGCTCAGGTTTGTCCAGAA
m-NQO1	CGTAGCAGGATTTGCCTACACA	GGCCAGAGAATGACGTTCATG
m-HO-1	CACAGATGGCGTCACTTCGT	TTGCCAACAGGAAGCTGAGA
m-IL-1β	CTTTCCCGTGGACCTTCCA	CTCGGAGCCTGTAGTGCAGTT
m-IL-6	ACCACTCCCAACAGACCTGTCT	CAGATTGTTTTCTGCAAGTGCAT
m-TNF-α	ACAAGGCTGCCCCGACTAC	TGGGCTCATACCAGGGTTTG
m-H2AX	GCGGTGCTCGAGTACCTCACT	CCAGCAGCTTGTTGAGCTCCT
m-cGAS	AAGAGTTTCAAGAGCTGGATGCA	GGCACTCAAGAAAGAATGCTAACA
m-STING	GGCTGGCCTGGTCATACTACA	CCCCACAGTCCAATGGAAAG
m-GAPDH	GCCACCCAGAAGACTGTGGAT	GGAAGGCCATGCCAGTGA

### 2.13 *In vivo* imaging analysis

To evaluate the localization of RA nanomicelles *in vivo*, we prepared RA nanomicelles loaded with DIR dye and administered them orally to mice. Fluorescence distribution in mice was analyzed using *in vivo* imaging systems (IVIS, Lumina XRMS, PerkinElmer, USA) (10 min, 30 min, 1 h, 3 h, 5 h and 8 h). To further investigate the tissue distribution of nanomicelles, mice were euthanized at 2 h using carbon dioxide, and visceral tissues such as heart, stomach, liver, spleen, lungs, and kidneys were collected. The fluorescence distribution in different tissues was analyzed using *in vivo* imaging system.

### 2.14 Statistical analysis

The statistical analyses were done by the Student's *t*-test, and comparisons of the means of 3 or more groups were performed using ANOVA with Tukey's post-hoc test using SPSS software. Differences with P < 0.05 were considered statistically significant.

## 3 Results

### 3.1 Preparation and characterization of RA-Myr nanomicelles

**Fig. 2:**
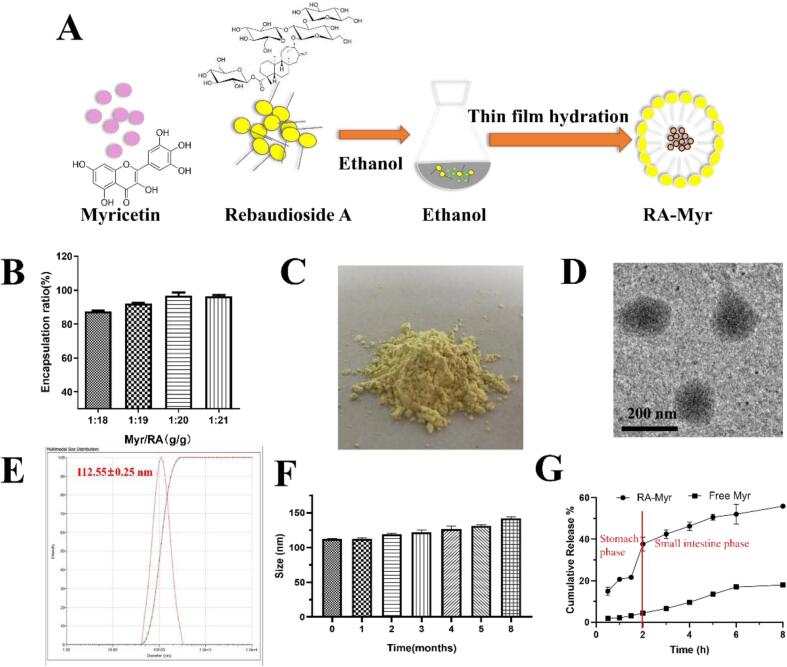
Preparation and characterization of RA-Myr nanomicelles. (A) Schematic diagram of the preparation process of RA-Myr nanomicelles. (B) Encapsulation efficiency (%) of Myr in RA-Myr at different mass ratios (Myr:RA = 1:18–1:21). Data: mean ± SD (*n* = 3). (C) Appearance of RA-Myr nanomicelles (Solid). (D) TEM image of RA-Myr nanomicelles (Bar = 200 nm). (E) The particle size of RA-Myr nanomicelles. (F) Stability of RA-Myr stored at room temperature over 8 months (size monitored monthly). (G) *In vitro* release profile of Myr from RA-Myr *vs.* free Myr suspension. Data are presented as the means ± SD.

The structure of RA contains hydrophobic diterpenes and hydrophilic sugar side chains, which can self-assembly into micelles in aqueous solution with a critical micelle concentration (CMC) of 5 mM (Mudgal et al., 2016). In this study, a series of RA-Myr nanomicelles with different mass ratios (Myr: RA = 1:18–1:21) was prepared as described in Fig. 2A. As shown in Fig. 2B, as the RA mass ratio increased, the encapsulation efficiency increased. When the Myr/RA mass ratio was 1:18, the encapsulation efficiency of Myr was 87.55 ± 0.37 %, and when the mass ratio was 1:20, the encapsulation efficiency was 96.73 ± 1.55 %. The RA-Myr was a uniform and flowable light-yellow powder (Fig. 2C). The TEM results showed that RA-Myr was uniformly dispersed in spherical or quasi spherical shape, without obvious aggregates (Fig. 2D). The particle size was 112.55 ± 0.25 nm (Fig. 2E), and the Zeta potential was 13.92 ± 1.06 mV. RA-Myr was prepared as a powder sample (Fig. 2C), and we conducted stability studies in this state. The results showed that the particle size of RA-Myr remained stable during an 8-month storage period (Fig. 2F). The results showed that the release of RA-Myr in artificial gastric juice (0−2*h*) was slow, while the release in artificial intestinal fluid was rapid and sustained. These results indicated that RA-Myr can be effectively absorbed in the intestinal tract (Fig. 2G). We also tested the ability of nanomicelles to pass through the intestinal barrier *in vitro* using trans-well, the results indicated that nanomicelles could be detected through Caco-2 monolayer cells and in the trans-well chamber. Interestingly, this process was also inhibited by MβCD. MβCD, as an endocytosis inhibitor, significantly inhibited the uptake of nanoparticles (Fig. S1). MβCD is a non-specific inhibitor targeting caveolal mediated endocytosis (Yu et al., 2013). These results indicated that nanomicelles can be absorbed into the bloodstream through the intestinal barrier.

### 3.2 *In vitro* antioxidant assay

**Fig. 3:**
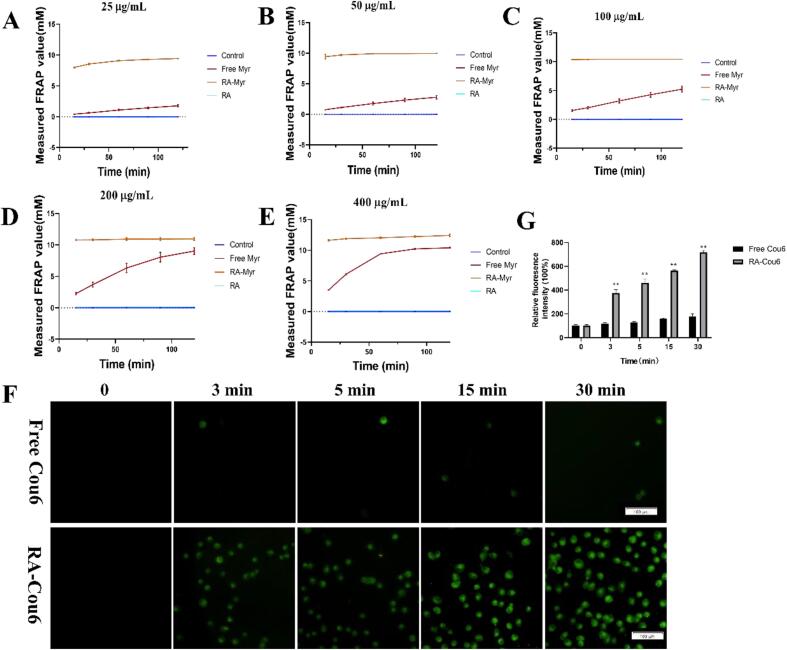
*In vitro* antioxidant activity and cellular uptake. (A-E) The antioxidant activity of free and RA-Myr nanomicelles was detected using the FRAP assay (incubation: 0–120 min; concentrations: 25–400 μg/mL). (F) Cellular uptake of RA nanomicelles. HK-2 cells were treated with free Cou6 or RA-Cou6 for 3, 5, 15 and 30 min, and photographed under a fluorescence microscope. (Bar = 100 μm). (G) Quantified fluorescence intensity of Cou6 uptake. Data are presented as the means ± SD. **P < 0.01 *vs.* Cou6 group.

As shown in Fig. 3A-E, the free RA solution exhibited no detectable antioxidant activity. While free myricetin (Myr) demonstrated concentration- and time-dependent antioxidant effects, the RA-Myr nanomicelles significantly enhanced antioxidant activity compared to free Myr (*P* < 0.01). The enhanced antioxidant activity of RA-Myr is likely attributable to the markedly improved dispersity of Myr facilitated by the RA-based delivery system. Specifically, the RA carrier increased the aqueous dispersity of Myr by 53-fold (Fig. S2), which corresponds with the significantly elevated antioxidant performance observed *in vitro*, underscoring the critical role of solubilization in enhancing bioactivity.

### 3.3 Cellular uptake

As shown in Fig. 3F-G, significant green fluorescence was observed in the RA-Cou6 group after incubation for 3 min, while weak fluorescence was observed in the free Cou6 group (incubation for 30 min), indicating that the RA-Cou6 delivery system improved drug uptake. The promoting effect of RA delivery system on cellular uptake may be beneficial for the expression of drug's biological activity.

### 3.4 RA-Myr nanomicelles protect cisplatin induced HK-2 cell damage *in vitro*

**Fig. 4:**
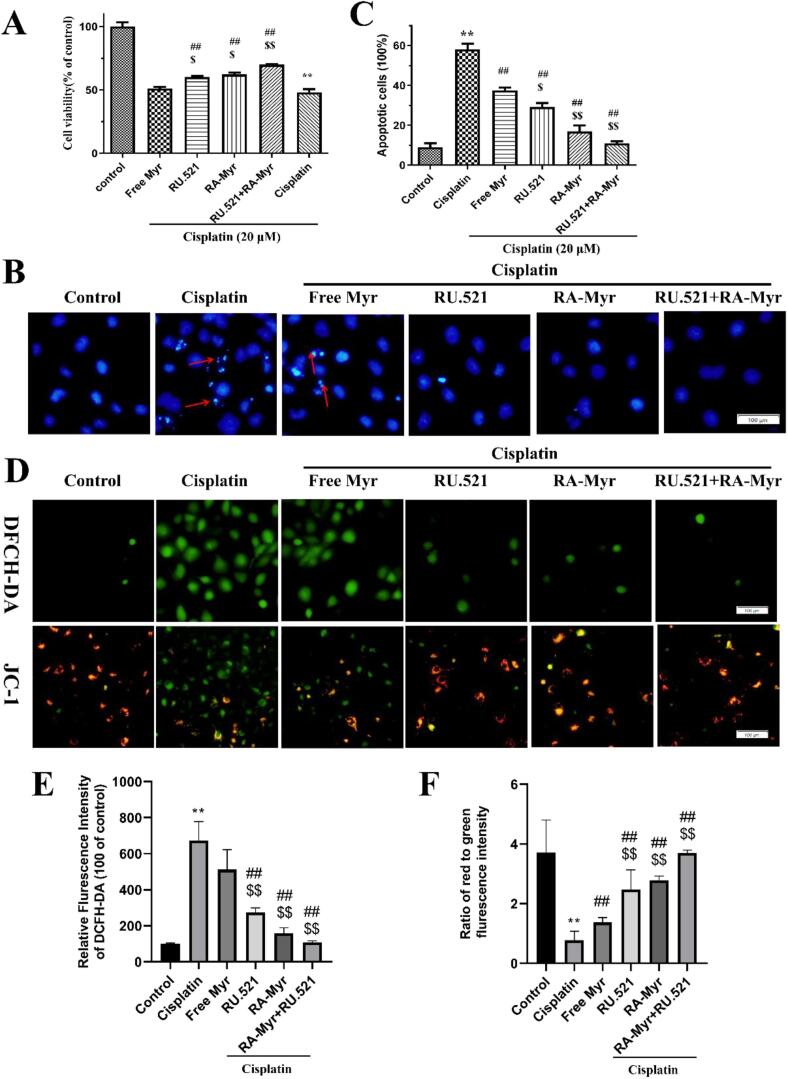
The *in vitro* protective effect of RA-Myr nanomicelles on cisplatin induced HK-2 cell damage. (A) Cell viability was measured using MTT assay after treatments: control, cisplatin (20 μM), free Myr, RA-Myr, RU.521 (cGAS inhibitor), or RA-Myr + RU.521. (B, C) DAPI staining showing apoptotic bodies (red arrows) in cisplatin-treated cells. (D) RA-Myr nanomicelles reverse cisplatin induced ROS production and MMP disruption. (Bar = 100 μm). (E, F) Analyze the fluorescence intensity of DCFH-DA and JC-1 results separately. Data are presented as the means ± SD. **P < 0.01 *vs.* control group; ##P < 0.01 *vs.* cisplatin group; &*P* < 0.05 and &&P < 0.01 *vs.* free Myr group. (For interpretation of the references to colour in this figure legend, the reader is referred to the web version of this article.)

As shown in Fig. 4A, cisplatin (20 μM) significantly inhibited the proliferation of HK-2 cells with an inhibition rate of 51.74 ± 4.13 %. The inhibition rate of the free Myr group was 48.79 ± 2.64 %, without any protective effect. Interestingly, RU.521 (an effective selective cGAS inhibitor) also alleviated cisplatin induced proliferation inhibition of HK-2 cells, and the combined use of RA-Myr nanomicelles and RU.521 had a more significant protective effect.

As shown in Fig. 4B and C, cisplatin induced morphological changes in HK-2 cells, such as apoptotic bodies. RA-Myr nanomicelles, RU.521, and their combined application all protected the aforementioned morphological damage induced by cisplatin. These results demonstrated the *in vitro* kidney protective effect of RA-Myr nanomicelles.

### 3.5 RA-Myr nanomicelles reverse cisplatin induced ROS production and MMP disruption

DCFH-DA and JC-1 probe labeling was used to detect intracellular ROS and MMP, respectively. Fig. 4D-F showed that cisplatin significantly induced the ROS accumulationand MMP disruption in HK-2 cells, Both RA-Myr nanomicelles and RU.521 inhibited cisplatin induced ROS production and MMP disruption, and their combination showed more significant effect.

### 3.6 RA-Myr nanomicelles protect against cisplatin induced DNA damage

**Fig. 5:**
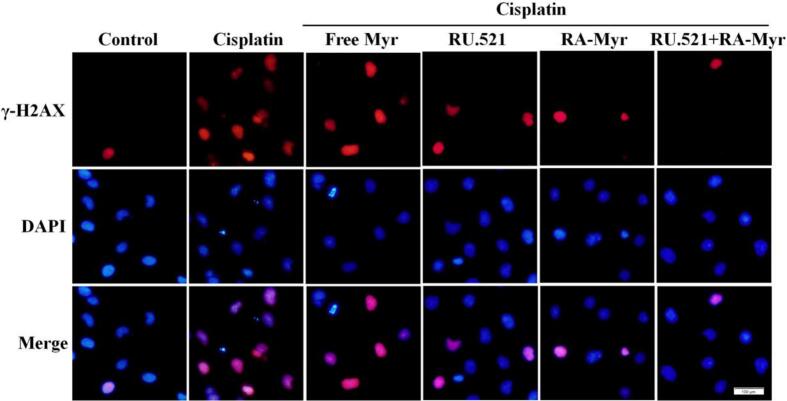
RA-Myr inhibits cisplatin-induced DNA damage. Immunofluorescence staining of γ-H2AX (DNA damage marker, red) in HK-2 cells. Nuclei: DAPI (blue). (Bar = 100 μm). (For interpretation of the references to colour in this figure legend, the reader is referred to the web version of this article.)

Immunofluorescence was used to detect DNA damage induced by cisplatin. Fig. 5 showed that cisplatin induced DNA damage in HK-2 cells, manifested by increased expression of γ-H2AX (a DNA damage marker). The expression of γ-H2AX in the RA-Myr nanomicelles and RU.521 group decreased significantly. Interestingly, the combination of the two showed a more significant decrease (Fig. 5). The above results demonstrated cisplatin induced DNA damage was inhibited by RA-Myr nanomicelles.

### 3.7 RA-Myr nanomicelles inhibit cisplatin induced activation of cGAS/STING pathway

**Fig. 6:**
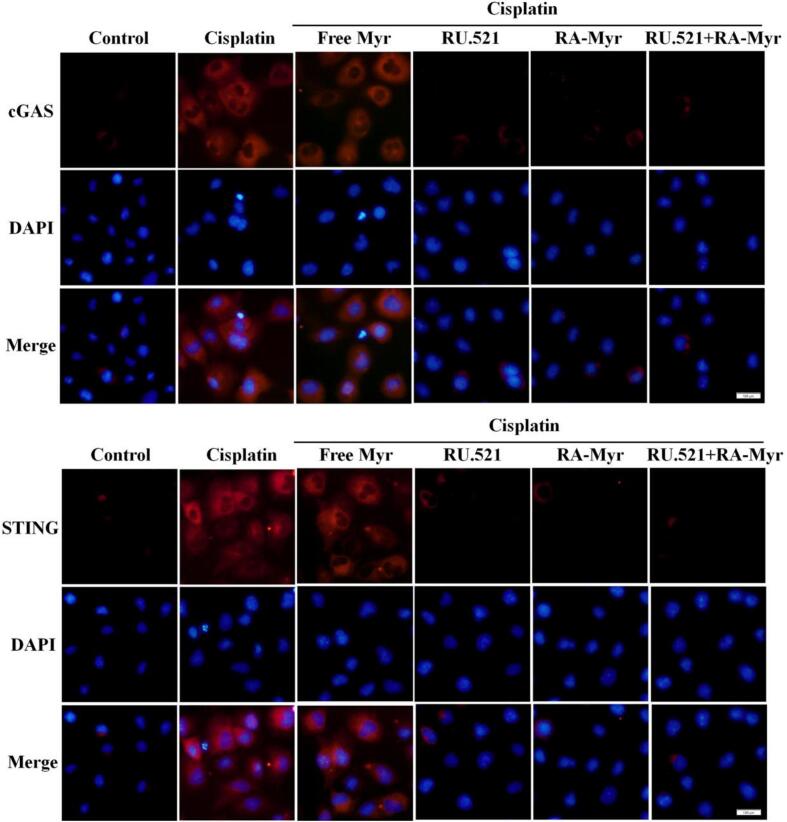
RA-Myr suppresses cisplatin-induced cGAS/STING pathway activation. Immunofluorescence of cGAS and STING (red) in HK-2 cells. (Bar = 100 μm). (For interpretation of the references to colour in this figure legend, the reader is referred to the web version of this article.)

The above studies demonstrated cisplatin induced DNA damage was inhibited by RA-Myr nanomicelles, which was of interest to us as studies have shown that the cGAS/STING signaling pathway was involved in cisplatin induced AKI. As shown in Fig. 6, the immunofluorescence results showed that the increase in cGAS and STING expression induced by cisplatin was inhibited by RA-Myr nanomicelles. Especially in the combined treatment group, where the fluorescence intensity decreased more significantly. These results indicated that the *in vitro* kidney protective effect of RA-Myr nanomicelles may be related to the cGAS/STING pathway.

### 3.8 RA-Myr nanomicelles protect cisplatin induced AKI *in vivo*

**Fig. 7:**
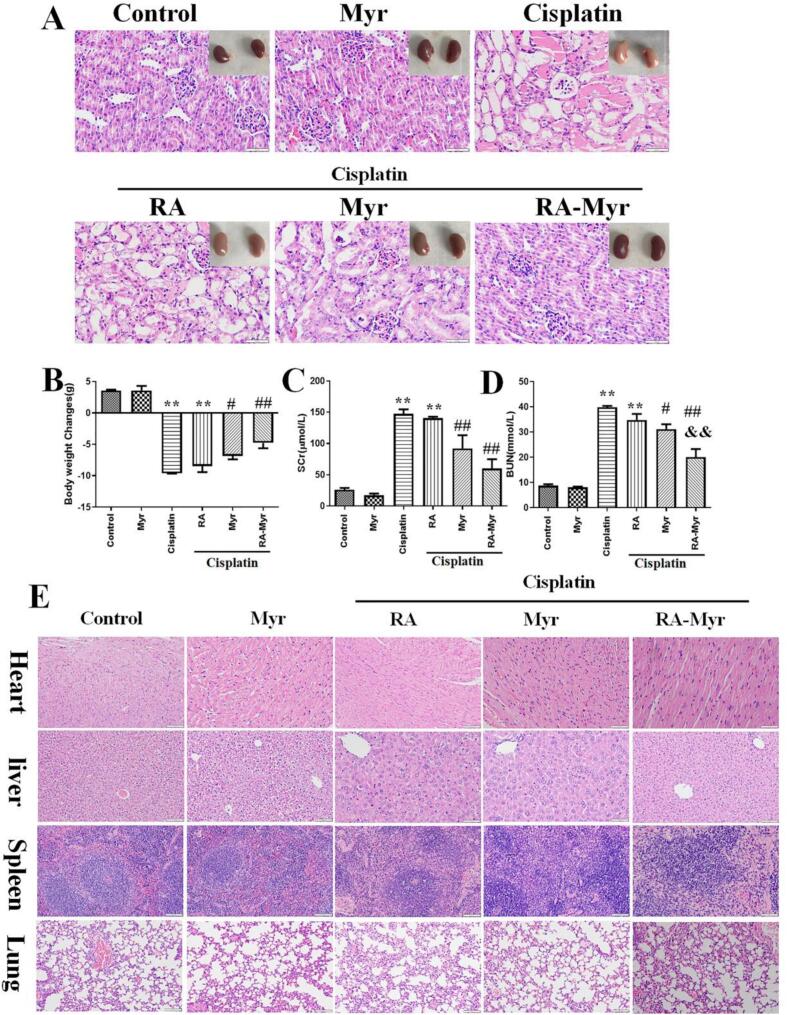
RA-Myr nanomicelles protect against cisplatin induced AKI. (A) Hematoxylin−eosin (H&E) staining (Bar = 200 μm) and kidney morphology observation. (B) Changes in body weight. (C) SCr levels. (D) BUN levels. (E) H&E staining of visceral tissues (heart, liver, spleen, lung). Data are presented as the means ± SD. **P < 0.01 *vs.* control group; #*P* < 0.05 and ##P < 0.01 *vs.* cisplatin group; &&P < 0.01 *vs.* free Myr group.

We established cisplatin induced AKI mice model *in vivo*. The mice were starved and intraperitoneally injected with cisplatin (20 mg/kg) 12 h later. The treatment group received oral Myr (100 mg/kg) or RA-Myr nanomicelles (100 mg/kg) 2 h before injection of cisplatin. The mice were treated with corresponding drugs for three consecutive days and then euthanized. The colour of the kidneys and the weight records of the mice showed pathological changes, whitening, increased edema, and significant weight loss in the cisplatin group (Fig. 7A and B). The above renal lesions and weight loss were significantly reduced in the Myr group and RA-Myr nanomicelle groups, and the symptoms were more significantly reduced in the RA-Myr nanomicelle group.

Pathological examination of kidney tissues were performed using H&E staining. As shown in Fig. 7A, compared with the control group, the mice in cisplatin group showed severe kidney damage, such as inflammatory cell infiltration and tissue vacuolization. Compared with the cisplatin group, the Myr and RA-Myr nanomicelle treatment groups reduced the aforementioned kidney damage induced by cisplatin, especially the RA-Myr nanomicelle group. Similarly, Fig. 7C and D showed the SCr and BUN levels were significantly increased after cisplatin treatment. Myr and RA-Myr nanomicelles treatment also reduced SCr and BUN levels, especially in the RA-Myr nanomicelles group. Mild, localized immune cell infiltration in liver and spleen was noted in RA and RA-Myr groups (Fig. 7E), likely reflecting RA-triggered immune activity. However, no functional impairment or systemic toxicity was observed. These results indicated the *in vivo* protective effect of RA-Myr nanomicelles.

### 3.9 RA-Myr nanomicelles inhibit DNA damage and cGAS/STING pathway

**Fig. 8:**
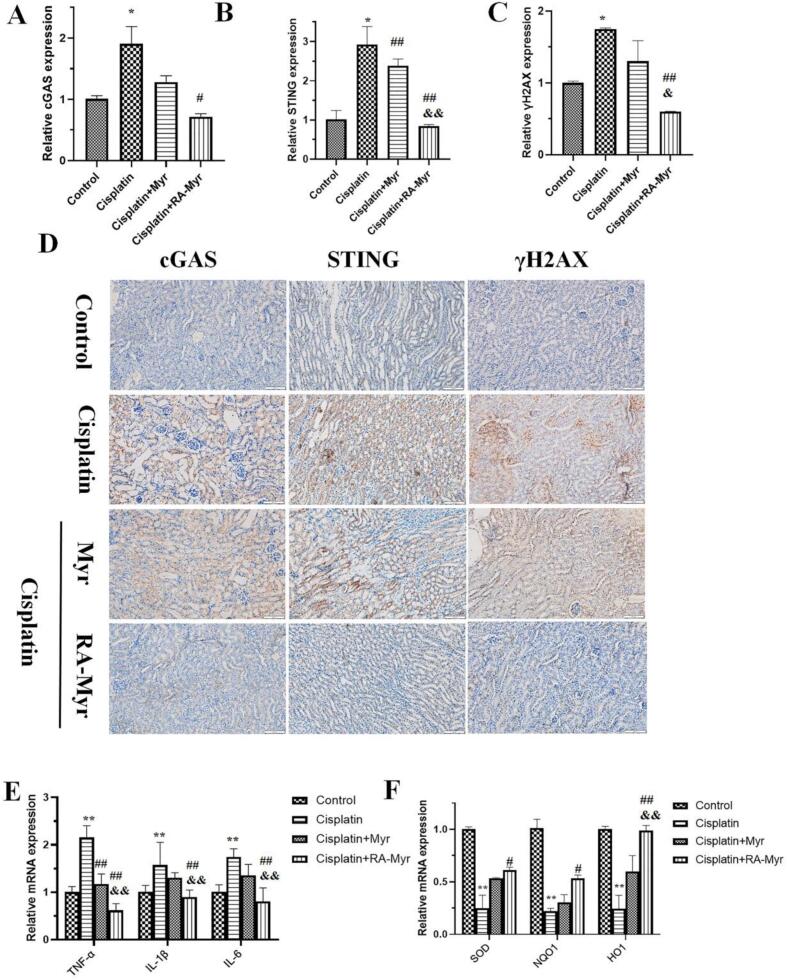
The mechanism of RA-Myr in protecting cisplatin induced AKI. (A-C) RT-qPCR detection of cGAS, STING and γH2AX. (D) Immunohistochemical detection of the expression of cGAS, STING and γH2AX. (E) RT-qPCR was used to detect the expression of inflammatory related factors TNF-a, IL-1, and IL-6. (F) RT-qPCR was used to detect the expression of antioxidant related factors SOD, NQO1, and HO-1. Data are presented as the means ± SD. *P < 0.05 and **P < 0.01 *vs.* control group; #P < 0.05 and ##P < 0.01 *vs.* cisplatin group; &P < 0.05 and &&P < 0.01, *vs.* free Myr group.

In order to explore the mechanism of renal protection in RA-Myr nanomicelles *in vivo*, RT-qPCR and immunohistochemistry were used to detect the expression of proteins related to the DNA damage cGAS/STING signaling pathway. Fig. 8A-8C showed that, both in the mRNA and protein level, the expression of γH2AX, cGAS, and STING in cisplatin group was significantly increased. Encouragingly, the expression of these three factors in RA-Myr nanomicelles group was significantly reduced.

As is well known, inflammatory response was also involved in cisplatin induced nephrotoxicity, which was closely related cGAS/STING pathway. The RT-qPCR results showed that the mRNA levels of inflammatory factors TNF-a, IL-1, and IL-6 in cisplatin group were significantly increased, while RA-Myr nanomicelle therapy alleviated the above elevated levels of inflammatory factors (Fig. 8E).

### 3.10 RA-Myr nanomicelles attenuates cisplatin-induced destruction of oxidative stress

It has shown that after cisplatin enters kidney tubular cells, it can cause inhibition of some antioxidant enzymes, such as SOD, inducing ROS generation, promoting DNA damage and cell apoptosis (Jesse et al., 2014). As shown in Fig. 8F, the mRNA levels of SOD, NQO1, and HO-1 in cisplatin group were inhibited (P < 0.01), and RA-Myr nanomicelles therapy significantly alleviated the inhibition of the aforementioned antioxidant enzymes induced by cisplatin.

### 3.11 *In vivo* imaging analysis

**Fig. 9:**
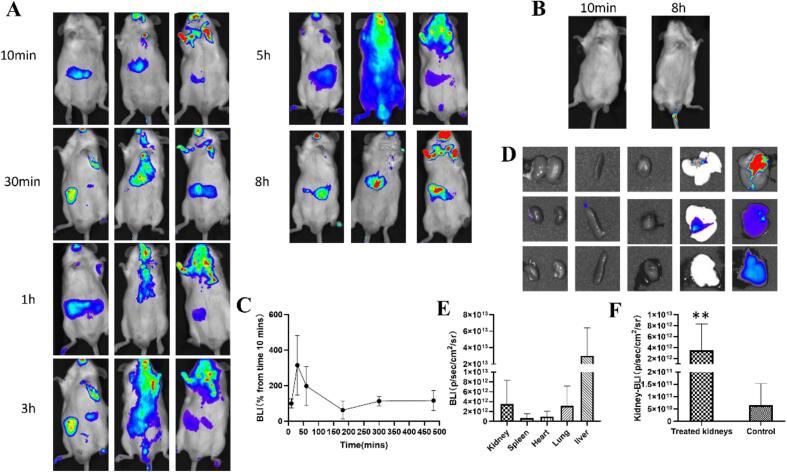
Biological distribution of RA/Myr DIR *in vivo* after oral administration for 0 to 8 h. (A) *In vivo* imaging of mice. (B) *In vivo* imaging of un-treated mice at 10 min and 8 h, (C) Comparison of abdominal luminescence intensity values of mice within 6 time points (based on 10 min). (D) *In vitro* images (2 h) of main tissues (heart, liver, spleen, lungs, kidneys). (E) Luminescence intensity values of mouse main tissues. (F) Statistical analysis of the fluorescence between the untreated control kidneys and the kidneys from mice treated with the RA/DiR nanomicelles. The values are expressed as mean ± SD (*n* = 3). **P < 0.01.

In order to investigate the distribution of RA nanodelivery systems *in vivo*, the NIRI system was used to study the visual biodistribution of DIR labeled RA delivery systems in mice. The results showed that drugs administered orally through the RA delivery system mainly accumulated in the liver. In addition, the distribution of drugs in the kidneys is also encouraging (Fig. 9A-E). The quantitative analysis (Fig. 9F) confirmed a statistically significant increase in fluorescence intensity in the kidneys of the RA/DiR-treated group compared to the untreated controls. This provided robust quantitative evidence to support the accumulation of the nanomicelles in the kidneys.

## 4 Discussions

Cisplatin is clinically used to treat various solid tumors, such as head and neck cancer, testicular cancer, ovarian cancer, lung cancer, *etc.* However, its serious adverse reactions, especially nephrotoxicity, are particularly prominent. Research has found that approximately 20–35 % of patients experience AKI after taking cisplatin (Zhang et al., 2022). There is a lack of effective treatment options for the nephrotoxicity of cisplatin. Repeated attacks of AKI can lead to impaired renal tubular function, acute renal failure, and sexual kidney disease, bringing serious physical and psychological burden to patients.

At present, there are no effective drugs or methods for treating AKI caused by cisplatin. Excitingly, research has found that many natural products such as flavonoids, saponins, alkaloids, *etc.* have shown potential for treating AKI (Cohen and Hunter, 2017; Lu et al., 2023; Ma et al., 2015). The latest research has found that flavonoids such as kaempferol, hesperidin, and formononetin exhibit promising potential in protecting cisplatin induced AKI (Chen et al., 2019; Hao et al., 2021; Shao et al., 2023). Myr is one of the most common natural flavonoids, widely present in berries and vegetables. Myr has excellent biological activities such as antioxidant, anti-inflammatory, hypoglycemic, and anticancer activities (Domitrovic et al., 2015; Huang et al., 2015; Yuan et al., 2015). Research has found that Myr plays a protective role in cisplatin induced nephrotoxicity. Although the development prospects of Myr are worth looking forward to, its poor water solubility leads to its low bioavailability *in vivo*, which limits its application. Free Myr is highly hydrophobic and demonstrates exceedingly low water solubility (<5 μg/mL in pure water). Since *in vitro* assays typically require compounds to be dissolved in aqueous media such as PBS or cell culture medium, free Myr is prone to aggregation or precipitation, thereby diminishing its effective concentration and bioavailability. One of the methods to solve the above problems is through nano-delivery systems (Qian et al., 2017; Yu et al., 2022). In our previous study, a Solutol HS 15 delivery system loaded with Myr was prepared and the kidney protective effect of Myr was enhanced (Qi et al., 2023).

RA is a steviol glycoside extracted from the natural plant *Stevia rebaudioides*. RA can self-assemble into nano micelles in aqueous solution, and previous studies have demonstrated its potential as a drug carrier for delivering insoluble drugs (Wang et al., 2021; Wang et al., 2020). Therefore, in this study, the natural product RA was used as a delivery carrier to construct an oral delivery system loaded with Myr, which can solve the problem of poor bioavailability of Myr. In this study, the prepared RA-Myr nanomicelles had a particle size of 112.55 ± 0.25 nm and excellent stability. In addition, the RA delivery system also increased cell uptake of drugs, which was beneficial for improving the biological activity of its loaded drugs. Micelles enhance the cellular uptake of hydrophobic drugs like Cou6 by improving solubility, preventing degradation, and utilizing active uptake mechanisms, thereby overcoming the limitations of free drugs despite their larger size. Moreover, the organizational distribution results were encouraging, and it must be pointed out that the accumulation of RA delivery system in the kidneys indicated its potential application value in the treatment of kidney related diseases. However, our study has not yet demonstrated the kidney targeting effect of RA nanomicelles, and further research and exploration are needed. Moreover, the glycoside structure of RA may contribute to biological activity and enhance therapeutic efficacy, but future studies using RA free controls (such as PEG/Myr) will decouple delivery from pharmacological effects and help elucidate the mechanism.

The mechanism of cisplatin induced kidney injury may include oxidative imbalance, induction of cell apoptosis, and inflammation (Maekawa et al., 2019b; Manohar and Leung, 2018). In addition, cisplatin mediates its cytotoxic effect by binding to DNA to form adducts that cause DNA damage, which is the main mechanism by which it exerts anti-tumor effects (Burns et al., 2021; Wang et al., 2024). Research has found that the sensitivity of cells to cisplatin is closely related to intracellular mitochondria (Yimit et al., 2019). The high mitochondrial density in the kidney makes them particularly sensitive to cisplatin, which may be the reason for cisplatin induced nephrotoxicity (Hu et al., 2020). On the other hand, mitochondria, as energy factories within cells, play an important role in regulating redox balance. Therefore, ROS scavengers and antioxidants exhibit strong protective effects on renal toxicity (Jiang et al., 2009). Our study showed that cisplatin induced the disruption of MMP and the accumulation of ROS and inhibited the proliferation of HK-2 cells. RA-Myr nanomicelles reverse cell death by alleviating cisplatin induced MMP damage and ROS accumulation. The *in vivo* studies also showed that RA-Myr nanomicelles alleviated cisplatin induced AKI and reversed the reduced expression of antioxidant enzymes such as SOD, NQO1, and HO-1 induced by cisplatin. Moreover, the *in vivo* results also indicated that RA-Myr nanomicelles treatment alleviated the expression of inflammatory factors such as TNF-a, IL-1, and IL-6 induced by cisplatin.

Moreover, cisplatin induced AKI is related to inflammatory reactions, which are caused by the activation of endogenous substances. As an intracellular DNA receptor, cGAS can recognize both exogenous DNA such as pathogen DNA and endogenous DNA such as mitochondrial DNA (mtDNA) and nuclear DNA (Bai and Liu, 2021; Decout et al., 2021). Activated cGAS activates STING, leading to type I IFN response and ultimately activating innate immune response (Ablasser et al., 2013; Gao et al., 2013). It has been reported that cGAS/STING signaling is associated with various pathogenic processes, such as tumors, liver injury and kidney injury (Kwon and Bakhoum, 2020; Mao et al., 2017; Oduro et al., 2022). The AKI induced by cisplatin is associated with DNA damage, mitochondrial dysfunction, oxidative stress, and inflammation (Manohar and Leung, 2018). The nuclear DNA damage induced by cisplatin is of interest to us by activating the cGAS/STING pathway. In this study, we investigated the effects of cisplatin on DNA damage. The results indicated that cisplatin induced the expression of γH2AX *in vivo* and *in vitro*, further activating the cGAS/STING pathway, while RA-Myr nanomicelles relieved cisplatin induced DNA damage and the activation of the cGAS/STING pathway. In addition, we found that the combination of RA-Myr nanomicelles and cGAS inhibitor RU.521 further inhibited cGAS/STING pathway. Our results indicated that the DNA damage- cGAS/STING signaling pathway was involved in cisplatin induced AKI, and RA-Myr nanomicelles exerted kidney protective effects by inhibiting DNA damage – cGAS/STING signaling. It is worth noting that there may be a correlation between oxidative stress and DSBs. Research has shown that excessive ROS can induce oxidative damage, including DNA strand breaks, DNA double strand aberrations, and other forms of DNA damage (Cline, 2012). It is interesting that increasing evidence suggests that DNA repair can stimulate ROS production and oxidative stress in turn. There are reports that UV induced DNA damage exhibits an increase in intracellular ROS levels as a stress response (Rowe et al., 2008). Therefore, studying the interaction between oxidative stress and DSBs will help advance drug development. Moreover, we focus on the activation of cGAS/STING pathway by cytoplasmic DNA. It is undeniable that cytoplasmic DNA is not the only factor activating cGAS/STING. Our research still has many deficiencies. We will continue to explore the kidney protection mechanism of RA-Myr nanomicelles in subsequent experiments, and the long-term biosafety and off-target effects of RA-Myr remain to be evaluated. While RA nanomicelles induced mild hepatosplenic immune activation, this did not translate to functional toxicity. Future studies will optimize RA dosing to minimize immune stimulation while preserving efficacy. We believe that studying the role of cGAS/STING signaling pathway in cisplatin induced AKI can provide a basis for prevention and treatment of kidney injury.

## 5 Conclusions

RA-Myr micelle system was established for delivering insoluble drug Myr. With a size of 112.55 ± 0.25 nm, RA-Myr nanomicelles enhanced the antioxidant activity and cellular uptake of Myr, as well as protecting HK-2 cells from cisplatin DNA damage. *In vivo* results also demonstrated the kidney protective effect of RA-Myr nanomicelles. Mechanism results indicated that the *in vitro* and *in vivo* kidney protective effect of RA-Myr nanomicelles might be related to the inhibition of DNA damage-cGAS/STING pathway. Although the current results cannot prove whether other pathways are involved in the renal protective effect of RA-Myr nanocapsules, our study at least provides a new perspective for exploring the protection drugs.

## Ethics declarations

The animal study was approved by the Qingdao University of Science and Technology Ethics Committee for Animal Experimentation (approval document no. QKDLL2024-10, Qingdao, China). The animal experiments in this research work complied with the ARRIVE guidelines and were conducted as per the U.K. Animals (Scientific Procedures) Act, 1986, and associated guidelines.

## CRediT authorship contribution statement

**Tian Wang:** Software, Methodology, Investigation, Data curation, Conceptualization. **Zhen Gao:** Validation, Software, Methodology, Formal analysis. **Chuanlong Guo:** Writing – original draft, Software, Methodology, Formal analysis. **Wenyong Zhu:** Writing – review & editing, Software, Investigation, Funding acquisition.

## Declaration of competing interest

The authors declare that they have no known competing financial interests or personal relationships that could have appeared to influence the work reported in this paper.

## Data Availability

No data was used for the research described in the article.

## Appendix A Supplementary data

Supplementary material Image 1
