# Supplementary material for: A novel nanomicelle based on Rebaudioside A: An oral nanoplatform with enhanced nephroprotective effect of myricetin
Source: Int J Pharm X. 2025 Sep 8;10:100389. doi: 10.1016/j.ijpx.2025.100389 (PMC12766103; doi:10.1016/j.ijpx.2025.100389)
Supplement: Supplementary file 1 — Supplementary material [file mmc1.docx]

**A novel nanomicelle based on Rebaudioside A: an oral nanoplatform with enhanced nephroprotective effect of myricetin**


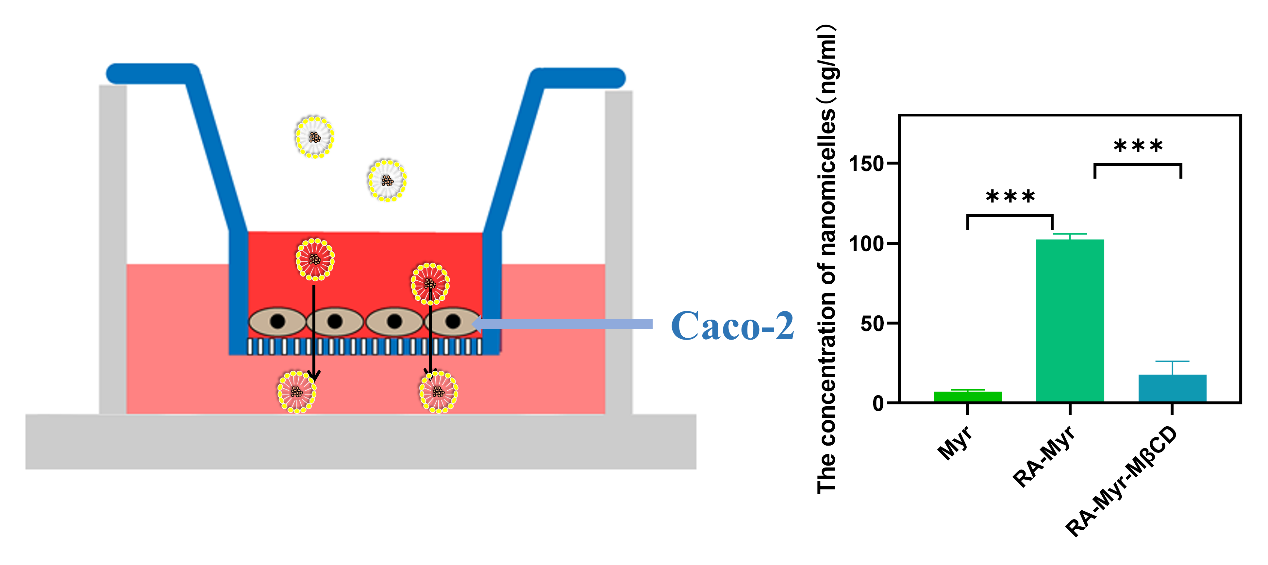


Figure S1. The ability of nanomicelles to cross the intestinal barrier in vitro was tested by Transwell.

Figure S2. The solubility of RA Myr and free Myr under different conditions.
